# Hot Sliding Wear of 88 wt.% TiB–Ti Composite from SHS Produced Powders

**DOI:** 10.3390/ma14051242

**Published:** 2021-03-05

**Authors:** Rahul Kumar, Le Liu, Maksim Antonov, Roman Ivanov, Irina Hussainova

**Affiliations:** Department of Mechanical & Industrial Engineering, Tallinn University of Technology, 19086 Tallinn, Estonia; rahul.kumar@taltech.ee (R.K.); le.liu@taltech.ee (L.L.); maksim.antonov@taltech.ee (M.A.); roman.ivanov@taltech.ee (R.I.)

**Keywords:** sliding wear, TiB-based composite, high temperature, SHS, SPS

## Abstract

Titanium alloys and composites are of great interest for a wide variety of industrial applications; however, most of them suffer from poor tribological performance, especially at elevated temperatures. In this study, spark plasma sintering was utilized to produce a fully dense and thermodynamically stable TiB–Ti composite with a high content of ceramic phase (88 wt.%) from self-propagating high temperature synthesized (SHS) powders of commercially available Ti and B. Microstructural examination, thermodynamic assessments, and XRD analysis revealed the in situ formation of titanium borides with a relatively broad grain size distribution and elongated shapes of different aspect ratio. The composite exhibits a considerable hardness of 1550 HV30 combined with a good indentation fracture toughness of 8.2 MPa·m^1/2^. Dry sliding wear tests were performed at room and elevated temperature (800 °C) under 5 and 20 N sliding loads with the sliding speed of 0.1 m·s^−1^ and the sliding distance of 1000 m. A considerable decline in the coefficient of friction and wear rate was demonstrated at elevated temperature sliding. Apart from the protective nature of generated tribo-oxide layer, the development of lubricious boric acid on the surface of the composite was wholly responsible for this phenomenon. A high load bearing capacity of tribo-layer was demonstrated at 800 °C test.

## 1. Introduction

Growing demand for titanium (Ti) and titanium alloys for a wide variety of industrial applications including, but not limited to, aerospace, marine, automotive, and biomedical industries have made Ti-based materials highly important. An extensive use of titanium alloys has been connected to their high strength-to-weight ratio, toughness, and extraordinary corrosion resistance. However, insufficient tribological performance of many alloys has hindered even wider applications of titanium, its alloys, and Ti-based composites [1,2], especially at elevated temperatures. To fill the gap and overcome the associated limitations, several methods including surface modifications [3], hard and soft coatings deposition [4], incorporating self-lubricating mediums [5], and reinforcements inclusion [6] are continuously reported. Titanium monoboride (TiB) is one of the most used additives for Ti matrix to yield improvement in mechanical as well as tribological properties due to Ti and TiB thermodynamic and chemical stability, similar density (4.5 g/cm^3^ for Ti and 4.56 g/cm^3^ for TiB), close values of coefficient of thermal expansion (8.2 × 10^−6^ °C^−1^ for Ti and 6.2 × 10^−6^ °C^−1^ for TiB), ability to retain protective tribo-oxide layer formed at elevated temperatures, and no spurious interfacial phase formation [1,7]. In situ formation of TiB during the reaction between Ti and TiB_2_ has drawn considerable interest and is widely studied [3,8]. The Gibbs free energy for the reaction (i.e., Ti + TiB_2_ → 2TiB) is a negative one (ΔG ~ −154 kJ/mol) demonstrating thermodynamical instability of TiB_2_ and Ti and, therefore, a high potential to form a more stable TiB [8]. At elevated temperatures and oxidative environment, Ti–TiB composites are suspected for the development of a dense rutile dominated oxides with a strong interfacial cohesion over the composites preventing the materials from severe oxidation and brittle fracture of sub-surface tribo-layers [9].

Changes in surface reactivity at elevated temperatures is a common attribute in metals. A large number of applications depends upon the ability of contacting surfaces to self-lubricate and/or to form protective oxide layers as a result of reactions with environments or during the tribo-contact [2]. Unlike steels and few others, oxides of Ti and its alloys are termed to be unprotective and undergo easy spallation during tribo-cycles [10,11]. Large lattice mismatch between the developed oxides and pristine Ti (or alloy) and ineffective Pilling–Bedworth ratio (P-B ratio) are the reason for such behavior [12]. Oxide scales developed over Ti and Ti–6Al–4V are reported to experience scaling, delamination, and constant fragmentation of brittle TiO, TiO_2_, and V_3_O_4_ [12,13], especially at temperatures above ~700 °C. Sliding wear tests accomplished on Ti–6Al–4V alloy under 10–50 N load at a room temperature were carried out with the integration of TiO_2_, Fe_2_O_3_, or mixtures of nano-scaled oxides onto sliding pathways, and the wear-increasing function was credited to TiO_2_ with a low load-carrying capability [11]. On the other hand, in [14], a thermal oxidation treatment of Ti6Al4V-10 vol.%TiC composites at a temperature of 600–800 °C was performed, revealing the development of a uniform oxide layer of rutile. Besides, a higher load carrying capacity of thermally oxidized composites was reported after a sliding test with 10 N of a load. Similarly, in [15], the development of a dense rutile dominated layer with a strong interfacial cohesion over Ti-8 vol.% TiB composites at 650 °C was demonstrated. The performance of the tribo-oxide layer was found to be greatly influenced by temperature, sliding velocity, and the load applied. In general, the lack of clarity regarding the properties and the elevated temperature wear performance of Ti–TiB composites in conditions of dry sliding has motivated this study.

Energy and time efficient spark plasma sintering (SPS) is one of the successfully used processing techniques to produce dense Ti-matrix composites with minimal porosity and tremendous improvements in mechanical as well as tribological properties [1]. It is a well-established fact that the successful performance of composite materials at service requiring movement of one part over another is attainable with the help of careful selection of constituents, manufacturing technology, and post-treatment. The method of the precursor powders preparation and developed microstructure are shown to be the main features for the Ti–TiB cermet as the composite for tribological applications.

Most of the time, the composites are consolidated using commercially available and conventionally mixed/milled precursor powders of Ti and TiB_2_, which often result in inhomogeneous structures with whisker-like TiB grains. To minimize these drawbacks, our approach proposes utilization of self-propagating high-temperature synthesis (SHS) or combustion synthesis for fabrication of the powder feedstock representing a collection of “pomegranate-like” particles consisting of TiB particulates bonded/clued by titanium. SHS has been widely accepted for production of various materials including ceramics, composites, and intermetallic and nano-phase materials [16,17] due to its high efficiency, negligible energy consumption, short processing time, and high productivity. It utilizes the advantages of extreme heat generated by the exothermic reaction in a reactive mixture during processing, resulting in the formation of a needed solid product [6]. Besides, SHS represents an in situ processing for the preparation of composite materials with interpenetrating phases of tailored composition.

As the formation of glazed layers of compacted oxides can provide wear protection to the composites at elevated temperatures [18,19], the current research work reports on the development of a tribo-layer during the sliding wear of spark plasma sintered Ti–TiB cermet of only 12 wt.% metal content, which were fabricated from the SHS produced “pomegranate-like” powders of Ti and TiB. Wear of the composite has been studied at the conditions of dry sliding at room (20 °C) and elevated (800 °C) temperatures. The responsible wear mechanisms have been evaluated, and the role of surface layer formation during elevated temperature sliding has been discussed.

## 2. Materials and Methods

### 2.1. Self-Propagating High-Temperature Synthesis

**Figure 1:**
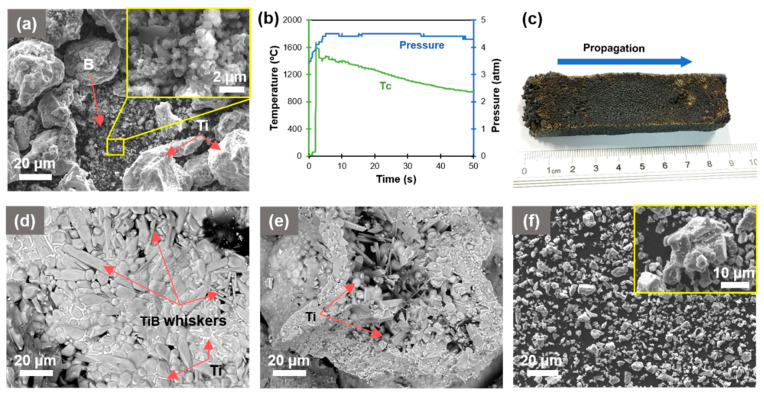
(**a**) SEM image of initial Ti and B powder mixture; (**b**) combustion temperature and pressure evolution; (**c**) high temperature synthesized (SHS) bulk product; (**d**,**e**) typical examples of SHSed fragments after manual crushing; and (**f**) composite powder after disintegrator milling.

The commercially available pure boron, B (purity >95%, Sigma-Aldrich, Taufkirchen, Germany, particle size <1 µm), and Ti (purity >99.5%, Alfa Aesar, Karlsruhe, Germany, particle size <44 µm) were prepared as 15 and 85 wt.% powder mixture, Figure 1a, for the combustion synthesis of the precursor suitable for SLM processing. The composition was designed to produce 85 wt.% of TiB with 15 wt.% of Ti upon a complete reaction xTi + yB → yTiB + (x − y)Ti. A mixture of reactants was homogenized in a ceramic mortar for 15 min and filled in a steel boat to prepare the rectangular green bodies of 10 mm length, 20 mm width, 15–20 mm height, and 50 grams’ weight. The samples were placed into a reaction chamber CPR-3.5l filled with argon gas of 99.999% purity and subjected to the pressure of 0.3 MPa. To initiate the combustion reaction, a 7 sec annealing of a tungsten coil positioned on the bottom side of samples was employed under 12 V. The mixture of KNO_3_ and Si powders with the weight ratio 1:4 was used as ignition agents. Two C-type tungsten-rhenium thermocouples (wire diameter 0.1 mm) preliminarily covered with a thin layer of boron nitride were positioned at holes drilled in the green body to record temperature-time profiles of the combustion process. Subsequently, the SHSed samples were crashed by a roller, then treated by disintegrator milling to make fine “pomegranate-like” powders, as detailed in [8]. The “pomegranate-like” powder represents the collection of composite particles with a relatively low size distribution and of the same flowability. Ceramic particles have been already embedded into the metallic titanium, which make the process of sintering much easier and more reliable.

The maximum combustion temperature (Tc) of 1700 °C was recorded. The computed average value of combustion velocity (Uc) is 2.12 mm/sec. The standard error of measurement for Tc and Uc were ±20 °C and 5%, respectively. The evolution of temperature (Tc) and pressure during synthesis is demonstrated in Figure 1b.

### 2.2. Spark Plasma Sintering

Prior to SPS processing, the powder blends were milled in a semi-industrial milling system DSL-115 disintegrator (TalTech, Tallinn, Estonia). The SHS synthesized Ti-B powdered product was consolidated using SPS (FCT Systeme GmbH, Frankenblick, Germany) at a temperature of 1200 °C with application of 50 MPa pressure and continuous direct electric current during 3 min in a vacuum. The mixture was loaded into a graphite mold (diameter—25.4 mm) and heated up (heating rate—100 °C/min) to the temperature of processing.

### 2.3. Materials Characterization

The produced cylinders with a thickness of 5 mm were refined to 1 µm finish with a Phoenix 4000 *(Buehler)* with the help of 8 inch diamond grinding discs (DGD Terra, *Buehler,* Esslingen, Germany). The polishing was executed with a speed of 300 rev/m during 3 min for each disc. The discs were changed in a sequence (74 µm, 40 µm, 20 µm, 10 µm, 3 µm, 1 µm) and water was used as a polishing medium. Then the polished specimens were cleaned by acetone and ethylene alcohol.

Microstructural inspection of the composites was made on polished and fractured surfaces using a high-resolution scanning electron microscope, SEM (HR-SEM Zeiss Merlin, Oberkochen, Germany) equipped with an energy dispersive spectrometer (EDS). The particle size distribution was assessed with the help of a laser diffraction using Malvern Mastersizer 3000, Almelo, Netherlands.

The chemical compositions of the powder mixture and the bulk samples were analyzed with the help of X-ray diffractometer (XRD, Siemens Bruker D5005 X-ray analyzer with a Philips X’Pert PRO diffractometer, PANalytical, Almelo, Netherlands) using Cu Kα radiation (λ = 0.1542 nm, 30 mA, 40 kV) with a scan step size of 0.02° and a count time of 0.4 s at each step. Relative fraction of the presented phases was estimated with the help of the Rietveld refinement method, which was performed by quantitative analysis of the crystalline phases detected by the corresponding XRD pattern.

The bulk density of composites was measured by means of the Archimedes’ principle (Mettler Toledo ME204 balance, Greifensee, Switzerland) with 0.1 mg accuracy and distilled water as the immersing environment. The reported density is an average of at least 3 measurements. The relative density calculations are based on the assumption of presence of phases detected by XRD and their volume fraction.

The bulk Vickers hardness (HV30) was estimated using a tester (Indentec 5030 SKV, Stourbridge, West Midlands, UK) at the indentation load of 30 kg applied for 10 s. The reported values are the mean of at least 5 indentations.

The indentation fracture toughness (IFT) was calculated from the length of radial cracks emanating from the corners of the indents following the Palmqvist approach [20]. The load of 50 kg·f on the indenter resulted in the quite well-developed crack systems, which mitigate any surface effects of the indentation. The length of the cracks initiated on the sample’ surface by the indent were recorded by optical microscopy (Zeiss Discovery V20, Oberkochen, Germany) equipped with AxioVision 4.8.2 software. For each sample, values of 5 recorded indents were averaged.

### 2.4. Sliding Wear Test

A universal tribo-test device CETR/Bruker—UMT-2 was employed for materials testing under dry unidirectional sliding in ball-on-plate configuration. The ball counterbody of Al_2_O_3_ with Ø10 mm (Redhill Precision, Prague, Czech Republic), hardness HV10 ≈ 1450, and surface roughness R_a_ = 0.02 µm was exploited under an applied load of 5 N (0.51 kgf) and 20 N (2.04 kgf), sliding speed of 0.1 m·s^−1^, and distance of 1000 m with the radius of a wear track of 4.5 mm. Each test consists of 20 equal periods with a duration of 500 s to assess friction progress during each test (start, running, and stop). An additional recalibration of device between periods was implemented to increase the test precision. This is especially important for the tests performed at elevated temperatures due to possible effect of a heat from a test chamber on the performance of force sensors positioned above it. The other parameters (load and frequency) were kept unchanged during the whole period. The duration of the test was selected to be sufficient to reach a steady state wear regime and to provide a measurable wear [21,22]. Surfaces of disk and ball were cleaned with ethanol, acetone, and then dried prior each test. Tests were conducted both at room and elevated temperature of 800 °C. The heating rate of 6 °C/min was settled to avoid unwanted thermal shock to test materials and equipment. The developed wear tracks were examined by SEM and evaluated by 3D profilometer Bruker Contour GT-K0+ to control the net missing volume.

The cross-section of the materials tested at 800 °C and both sliding loads of 5 and 20 N were examined with the help of SEM (HR-SEM Zeiss Merlin, Oberkochen, Germany) to explore the nature of the developed tribo-oxide layers.

In addition, oxidation tests were conducted in a laboratory furnace (Nabertherm muffle furnace, Lilienthal, Germany) at 300, 500, 700, and 800 °C to evaluate the oxide layer development. The oxidation duration was kept similar to the sliding test duration (10,000 s). The oxidized samples were checked for weight gain to the nearest 0.1 mg using a Mettler Toledo ME204 and surface Vickers microhardness (HV0.5) using a tester unit (Indentec 5030 SKV, Stourbridge, West Midlands, UK) applying 500 g (0.5 kg) load for a dwell time of 10 s. The load of indentation was selected to provide precise imprint diagonal measurement and to reduce the influence of the underneath material. The values reported are averaged for at least 5 indentations/measurements made.

## 3. Results and Discussion

### 3.1. Microstructural Analysis

Figure 1a,b show SEM images of the initial powder mixtures and the combustion thermogram and pressure during SHS.

According to the curve of temperature evolution during the combustion process, the temperature of 1700 °C is sufficient to melt Ti and dissolve or interact with B (according to phase diagram presented in [7]) to form TiB as per the principle of reaction diffusion. Additionally, an excess of Ti in the system serves as a matrix phase in a composite powder. It is to be noted that the high temperature during synthesis will not retain for a long time and a rapid drop in the temperature of the part below melting point of Ti (~1650 °C) is demonstrated (Figure 1b). A concurrent decrease in combustion temperature and an increase in pressure (due to sudden gas expanding from reaction frontier) creates the kinetic conditions which favored the formation of spherically shaped Ti droplets/particles.

The SEM images of a crashed SHS product in Figure 1d, e demonstrate the co-existing agglomerates of fine and coarse particles of either rounded or needle-like morphology together with spherical molten Ti droplets, as Ti was given with an excess amount to the initial mixture. The TiB whiskers mostly grow towards the center of the piece of material after SHS processing, which can be explained by the fact that elemental boron tends to be present in the center of segregation on subsequent precipitation from molten solution of Ti and B.

Figure 1f shows the SEM image of Ti–TiB composite powder after disintegrator milling. Particle size distribution (PSD) specified in Table 1 reveals the median diameter (D50) of Ti–TiB powder of around 10 µm.

**Figure 2:**
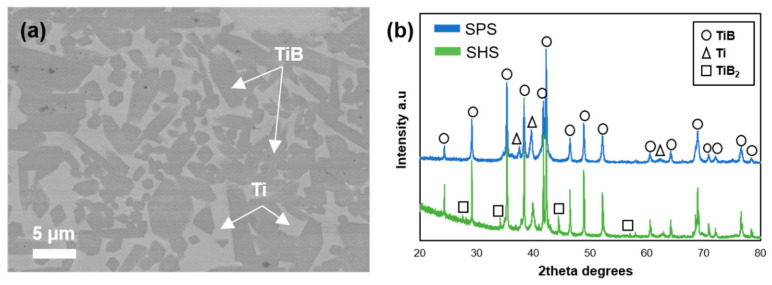
(**a**) SEM image of SPSed (spark plasma sintering) composite; and (**b**) XRD patterns of SHSed powder and SPSed composite.

The XRD patterns of SHSed Ti–TiB powder confirm the presence of TiB, TiB_2_, and free Ti as per Equation (1), which describes the first step of composite development, Figure 2. The presence of TiB_2_ phase together with Ti and TiB was also reported in [23,24], mainly due to either the inhomogeneity of B in the mixture powder or the insufficient time for completion of the reaction between TiB_2_ and Ti. The approximate concentration of 17.4% free Ti, 66.4% TiB, and 16.2% TiB_2_ was evaluated through the analysis of the XRD pattern of the SHSed powder, Figure 1f.

However, upon SPS, no evident TiB_2_ is identified by the XRD of the composite bulk as duration of the process allows complete chemical reaction between TiB_2_ and Ti as per Equation (2), which describes the second step of composite fabrication (SPS) resulting in complete reaction between available TiB2 and free Ti; therefore, the stable TiB phase and monoclinic α-Ti are the main constituents of the composite. The XRD pattern of SPSed bulk recorded from the polished surface confirms the concentration of TiB phase of ~87.8% and Ti phase of ~12.2% with neither TiB_2_ nor other phases.

Figure 2a demonstrates the SEM image of the composite revealing rather homogeneous distribution of TiB phase (grey) throughout the monoclinic Ti (white).

(1) $$\left(1+x\right)\mathrm{Ti}+1.5B=0.5{\mathrm{TiB}}_{2}+0.5\mathrm{TiB}+x.\mathrm{Ti}$$

(2) $${\mathrm{TiB}}_{2}+\left(x+1\right)\mathrm{Ti}=2\mathrm{TiB}+x.\mathrm{Ti}$$

The TiB–Ti composite produced by SPS from the conventionally milled/mixed commercially available powders of Ti and TiB_2_ are detailed in [19]. Fabrication of the fully dense composites with a high hardness of ~1324 HV30 and an indentation fracture toughness of 10.5 MPa·m^1/2^ has been reported. The homogeneous distribution of needle-like colonies of fine TiB was shown to be a key feature of the microstructure. The composites produced out from SHSed “pomegranate-like” powders display a relatively large distribution in the grain’ shape (from elongated with aspect ratio of 4 to equiaxed) as well as in the grain’ size (from as small as 0.5 µm up to as large as 10 µm), which, by very rough estimation, may be considered as bi-modal distribution. Tiny pores are only observed inside the large grains of TiB, indicating the high level of densification at the given conditions. A composite of only 12 wt.% Ti has been consolidated to 99.7% of relative density, suggesting an improved densification of the material of high ceramic content.

### 3.2. Mechanical Properties

**Figure 3:**
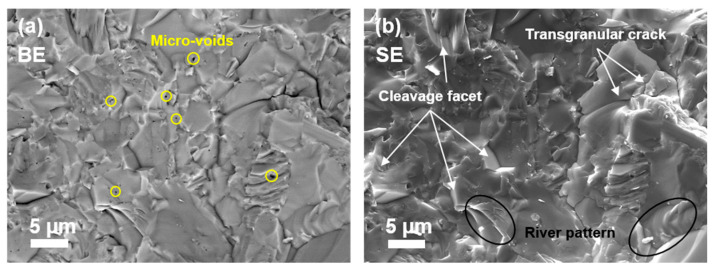
SEM images of fractured cross-sections: (**a**) Backscattered electron (BE); and (**b**) secondary electron (SE).

The density, hardness, and indentation fracture toughness (IFT) values for pure Ti and various Ti–TiB composites produced by different methods are listed in Table 2. Figure 3 demonstrates the images of fractured SPSed bulks.

The Vickers’ hardness of TiB is about 1800 HV and, therefore, the high hardness of the TiB rich composite in the current research is ascertained (Table 2). Apart from a high hardness, a good IFT value (8.2 MPa·m^1/2^) is demonstrated due to the presence of metallic Ti in the composite. In general, it is challenging to produce a composite material with a high content of ceramic phase (here TiB) showing a commendable IFT value, Table 2, as decrease in the IFT and density with an increase in ceramic content is widely reported. However, in the current study, a high relative density and IFT are evidenced even with an extremely high ceramic content.

The SEM image of fractured composite surface, Figure 3, demonstrates significant amount of plastic deformation and few cleavage facets/steps, which are characteristic of a granular fracture. In addition, the river pattern features of the cleavages are also noticed pointing at a brittle mode of fracture. Intergranular as well as transgranular crack propagation is one of the features of composite fracture. It appears that the crack is initiated at a point on the interface of TiB grain and propagated alongside this interface, consuming a large amount of energy, and thus enhancing the fracture toughness. The propagating crack, upon confronting a TiB particle, tends to encircle it and is deflected from its original track. Therefore, the crack deflection mechanism of fracture can be held responsible for improved composite toughening. A similar phenomenon was also reported in [7,25,26].

### 3.3. Sliding Wear

**Figure 4:**
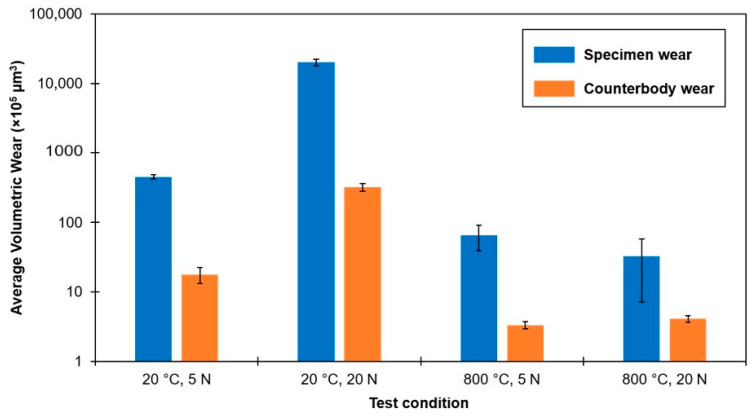
The effect of temperature and load on wear rate of Ti–TiB specimen and alumina counterbody (logarithmic scale).

**Figure 5:**
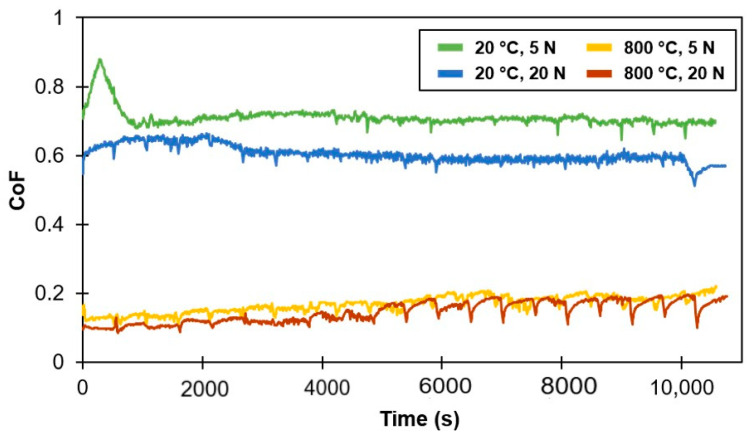
Effect of temperature and load on evolution of coefficient of friction.

**Figure 6:**
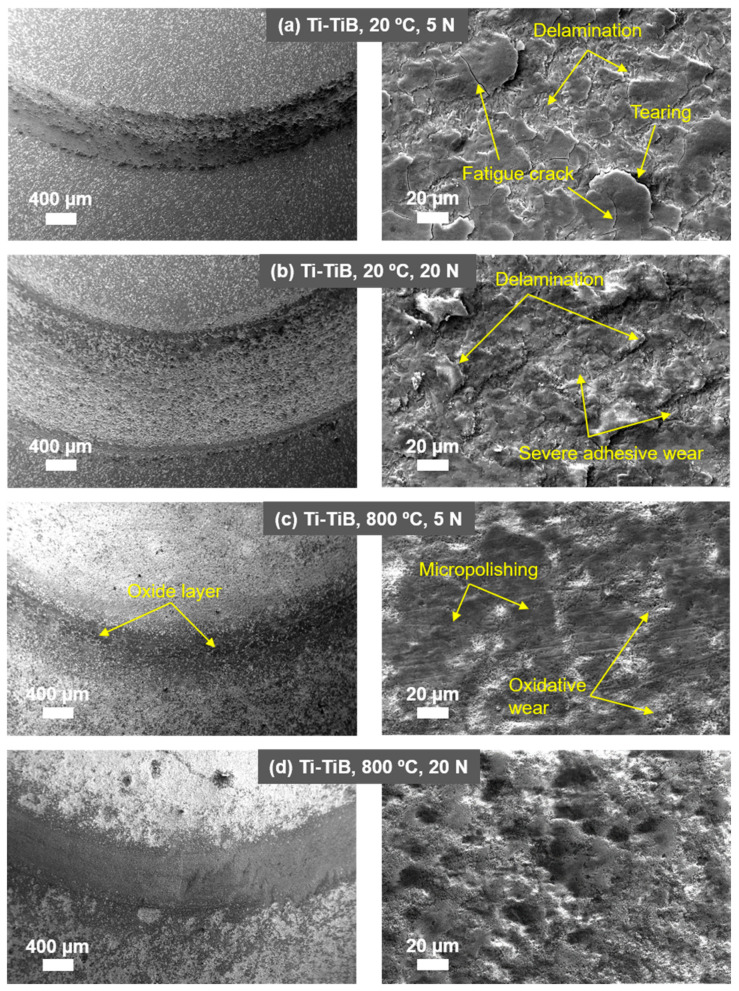
SEM images of composite surfaces with different magnification taken after sliding test. The test conditions are indicated in the figure.

**Figure 7:**
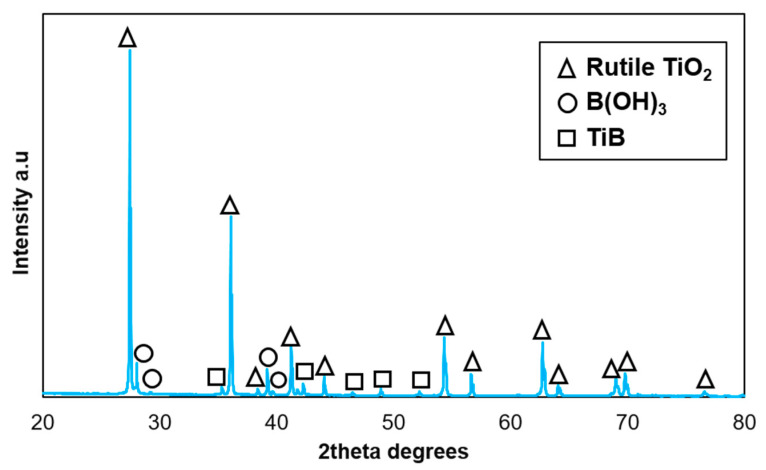
XRD pattern of composite surface after sliding at 800 °C, 20 N.

Figure 4 demonstrates the effect of temperature and load on the wear rates of the composite along with the counterbody ball. Figure 5 shows the coefficient of friction (CoF) evaluation during sliding tests. Figure 6 displays the scanning electron microscope (SEM) images of the worn surfaces. The XRD pattern of the composite after 800 °C sliding test is given in Figure 7.

The wear rates and CoF values of composite tested at 800 °C are significantly reduced in comparison to RT test (Figure 4 and Figure 5, Table 3). A distinct behavior of material at different load is clearly noticed. At a room temperature, an increase in load results in an expected rise in wear rate. However, at 800 °C, the wear rate is reduced with an increase in the test load.

The coefficient of friction is lower during tests performed at a higher load and/or temperature (Table 3, Figure 5). At a temperature of 800 °C, a lower CoF (during test with 20 N), as expected, resulted in a lower wear rate of material; while at room temperature, a lower CoF (during test with 20 N) resulted in a sufficiently higher wear rate of the Ti-TiB specimen. This can be explained by change in wear mechanism or the formation of new phases. The stabilization of CoF (reduction of fluctuations, running-in period) at a high temperature required significantly less time (100 s) as compared to the test at RT (3000 s), Figure 5. The range of CoF fluctuations and time to stabilization are influenced by properties of material, surface preparation (roughness, defects induced, presence of minor pollutants etc.), as well as mechanisms of tribo-layer formation. The running-in accompanied with formation of a characteristic tribo-layer is an essential process affecting the following steady state wear mechanism and the ultimate wear rate [27]. It is worth to note, that at 800 °C, a short running-in period was seen for both low and high sliding loads. It is important to mention that, at 800 °C, especially with maximum test load (20 N), the CoF is the lowest after restarting (Figure 5), and experiences insignificant growth during each sliding period. This leads to the conclusion that intervals between sliding-oxidation are favorable for tribo-couple. Table 3 specifies the CoFs measured at the end of the tests.

Figure 6 illustrates the features of the main wear mechanisms associated with the composite subjected to sliding under given conditions. The composite surface after RT sliding is dominated by the features, which are characteristic for adhesive and delamination wear modes. It is a well-established fact that a long-time sliding results in a higher strain level at the composite surface causing the development of fatigue-driven surface or sub-surface cracks [26], which grow to a critical length during the sequence of sliding cycles, followed by material delamination from the surface. This time-progressive process of delamination can also be observed in Figure 6a, where surface cracks progressively propagate towards other cracks causing detachment of a material from the surface. The detached wear debris at RT may further act as a third body intensifying the wear [28]. Apart from delamination, adhesion and abrasion are the common mechanisms of wear during RT sliding. At a higher load, the adhesion and abrasion mechanisms escalated, resulting in high wear of composite, which is characterized by deep plough grooves and almost doubled width (≈1 mm) of a wear track as compared to the test under 5 N load. In all probability, a flash surface temperature is sufficient to cause the material transfer (adhesive wear) from the composite surface to the counterbody. It is confirmed by the fact that a substantial amount of material transfer for RT sliding was encountered on the worn counterbody surface at both tests performed at 5 and 20 N. The transferred material mostly consisted of elements from composite surface, i.e., titanium and boron.

At 800 °C, an oxidative wear has demonstrated a noticeable effect. The frictional surface was dominated by an even oxide layer. In general, the wear at this stage is mainly conditioned by the fragmentation of oxide scales; the direct contact between the alumina ball and the composite surface is limited. The XRD pattern in Figure 7 details the phases on the surface demonstrating the formation of rutile and the presence of boric acid.

**Figure 8:**
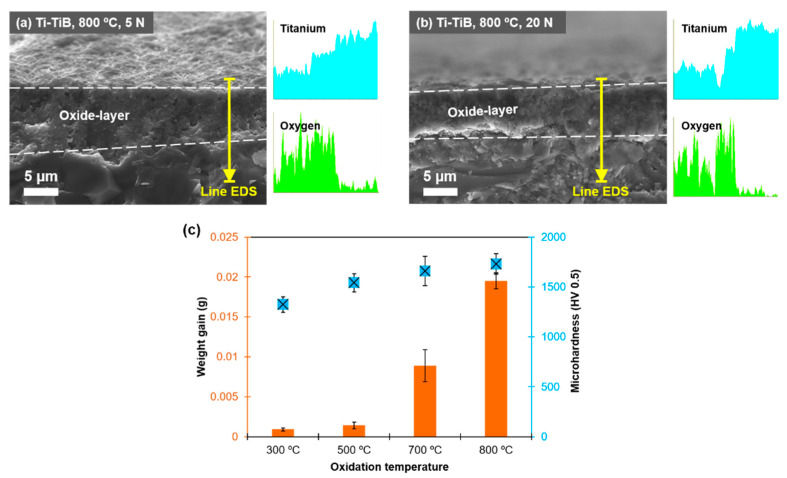
(**a**,**b**) SEM images of a central part of wear track cross-section of the composites tested at 800 °C with load of 5 or 20 N; (**c**) effect of oxidation temperature on weight gain and microhardness of composite.

During cross-sectional examination of the wear tracks on the surface of the composites tested at 800 °C, the development of quite thick (≈ 8 µm), uniform, and well-adhered to the parent material tribo-oxide layer was confirmed, Figure 8. The tribo-layer is thick enough to prevent direct contact between the composite surface and alumina counterbody. The oxide layer is worn out during successive sliding cycles, following by rapid re-oxidation of the exposed or damaged surfaces, preventing further contact between the parent surfaces [28]. No considerable difference in thickness and roughness of the developed surface layers inside and outside of the wear track was revealed. Therefore, the counterbody ball was borne by the generated tribo-oxide layer, and there is a low chance of complete removal of tribo-layer and sliding over the unprotected surface. Moreover, at elevated temperatures, the generated third body or retained debris are reported to undergo compaction and contribute to the development of a mechanically mixed layer responsible for a stable CoF and reduced wear rate [9,28]. However, the compaction of debris is much less pronounced at RT. Moreover, the wear is intensified by brittleness of original material, which can be easily fractured by wear debris retained in the wear scar. Apart from oxidation, micropolishing, which is commonly reported phenomenon under elevated and high temperatures sliding [19], can also be considered as one of the wear mechanisms.

Figure 8c demonstrates the effect of temperature on surface hardness and weight gain of Ti–TiB composite. All samples show negligible weight change at temperature below 500 °C, suggesting an insignificant oxidation evolution. The material exhibits a substantial weight gain and increased surface hardness at 700 and 800 °C due to the formation of rutile (Table 2). It is well documented that the first phase to form under oxidative media is anatase due to its lower surface energy and its further transformation into a harder and thermodynamically stable rutile at elevated temperature [29]. Therefore, it can be stated that the sliding behavior of composite at 800 °C is positively influenced by the rutile formation, which is a hard, thermodynamically stable, and thick layer of a P–B ratio in between 1–2 characterized by a higher load bearing capacity as compared to anatase.

Apart from rutile, the XRD pattern in Figure 7 reveals boric acid, B(OH)_3_, presented on the surface of the composite after sliding at 800 °C. The slippery or lubricating behavior of boric acid is detailed elsewhere [30] in imparting a low CoF due to its layered crystal structure. The occurrence of boric acid is conditioned by the oxidation of boron above 600 °C with formation of boric oxide, B_2_O_2_ as per Equation (3); and further B_2_O_2_ readily reacts with moisture in humid environment as per Equation (4). A surface film of a lamellar structure (as B(OH)_3_) consists of the atomic layers aligned parallel to the direction of sliding, causing them to easily slide over one another due to and interlayer slip [5].

(3) $$4B+3O_{2}=2B_{2}O_{3}$$

(4) $$\frac{1}{2}B_{2}O_{3}+\frac{3}{2}H_{2}O=B{\left(\mathrm{OH}\right)}_{3}$$

**Figure 9:**
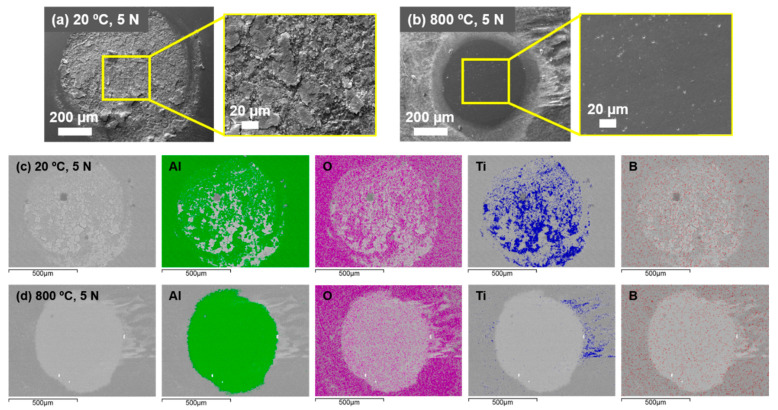
(**a**,**b**) SEM images of the counterbody ball worn surface; and (**c**,**d**) energy dispersive spectrometer (EDS) mapping of the worn area of the counterbody tested at 20 °C and 800 °C with load of 5 N.

Figure 4 demonstrates rather low wear of the counterbody alumina ball at the given sliding conditions. The hardness of alumina is comparable to that of the Ti–TiB composite (HV1450 vs. 1550, respectively); thus, the comparable wear rates of both bodies in contact are expected. Since, during an EDS study (Figure 9c,d), some amount of Ti–TiB specimen material transfer is evident on the Al_2_O_3_ ball surface. In all probability, the contact area of the ball should have higher temperature due to the fact that it is experiencing continuous friction, while the material of wear scar of the disk is experiencing wear only during passing of the ball and can slightly cool down between such passes.

More intensive transfer of titanium onto the counterbody in the case of RT sliding confirms the evidence of a severe adhesion between tribo-bodies. However, no detectable Ti transfer was recognized after the test conducted at 800 °C. It is understood that boron transfer from the composite to counterbody surface resulted in the formation of lubricious boric acid at elevated temperature sliding, thus minimizing counterbody as well as composite wear. At RT sliding, adhesion and abrasive marks were recognizable on the ball surface (Figure 9a), as the protection of oxide and lubricating boric acid layers is negligible. However, at 800 °C sliding under both loads, no marks of materials adhesion in the worn area were evidenced, Figure 9b. This was also confirmed by EDS analysis (Figure 9d). A smooth, polished surface of counterbody is clearly seen in Figure 9b.

## 4. Conclusions

In this study, a fully dense and thermodynamically stable TiB–Ti composite of TiB ceramic phase content of ~88 wt.% is densified by spark plasma sintering at a temperature of 1200 °C with the application of 50 MPa pressure during 3 min in vacuum. The “pomegranate-like” powder feedstock is produced with the help of self-propagating high-temperature synthesis of commercially pure boron and titanium at the combustion temperature of 1700 °C. During SPS, the TiB phase is mostly grown as equiaxed grains of a large size distribution. The bulks of TiB–Ti exhibit the hardness of ~ 1550 HV30 combined with a good indentation fracture toughness of 8.2 MPa·m^1/2^.

The wear behavior at room and elevated temperature of 800 °C under dry sliding conditions under loads of 5 and 20 N is shown to be highly affected by the temperature of the test. A considerable decrease in wear rate and the coefficient of friction is demonstrated at 800 °C. The protective behavior and a high load bearing capacity of generated tribo-oxide layer at 800 °C is confirmed. The significant decrease in the coefficient of friction (i.e., ~0.18) at elevated temperature is attributed to the generation of lubricious boric acid on the composite surface. Abrasion and adhesion are the main mechanism of wear at room temperature sliding, whereas micropolishing of oxide surface dominates at 800 °C.

## Figures and Tables

**Table 1:** Size distributions of the SHSed Ti–TiB powders.

Particle Size (Ti–TiB SHSed Powder)		
D10 (µm)	D50 (µm)	D90 (µm)
2.12 μm	8.98 μm	27.2 μm

**Table 2:** Comparison of processing methods, relative density, hardness and indentation fracture toughness of Ti-TiB composites.

Material	Process	Relative Density (%)	Hardness	IFT (MPa⋅m^1/2^)
CpTi [1]	SPS	97.92 ± 0.03	291 ± 10 (HV30)	-
TiB_w_-60 wt.% Ti [15]	Mixing + SPS	99.6%	-	9.35
TiB_w_-50 wt.% Ti [19]	Mixing + SPS	99.4%	1324 ± 18 (HV30)	10.52
TiB- 33 vol% Ti [24]	Ball Milling + Reaction hot pressing	-	1351 (HV50)	-
TiB–30 wt.% Ti [17]	SHS + PHIP (Pseudo Hot Isostatic Pressing)	98.45	87.8 HRA	6.15
TiB–20 wt.% Ti [17]	SHS + PHIP	97.57	86.7 HRA	5.23
TiB–12 wt.% Ti (current work)	SHS + SPS	99.7	1550 ± 26 (HV30)	8.16

**Table 3:** Effect of temperature and load on CoF measured at the end of test.

Test Conditions	Final CoF
20 °C, 5 N	0.71 ± 0.02
20 °C, 20 N	0.60 ± 0.02
800 °C, 5 N	0.19 ± 0.02
800 °C, 20 N	0.18 ± 0.02

## Data Availability

The data presented in this study are available on request from the corresponding author.
